# Molecular Mechanisms of DNA Damage Response and Epigenetic Regulation in Cold-Adapted Species: Implications for Genome Stability and Molecular Network Perspective

**DOI:** 10.3390/cimb47110923

**Published:** 2025-11-06

**Authors:** Olawale O. Taiwo, Waliu Alaka, Kenneth B. Storey

**Affiliations:** 1 Department of Biology, Institute of Biochemistry, Carleton University, 1125 Colonel By Drive, Ottawa, ON K1S 5B6, Canada; 2 Department of Biology, University of Western Ontario, 1151 Richmond Street, London, ON N6A 3K7, Canada

**Keywords:** freeze-tolerance, DDR, histone modifications, molecular network perspective, epigenetics

## Abstract

Cold-adapted species display remarkable genomic resilience under prolonged freezing and thawing cycles that would be lethal to most organisms. This review synthesizes current knowledge on the molecular mechanisms of DNA damage response (DDR) and epigenetic regulation that collectively safeguard genome integrity in these organisms. We highlight key DNA repair pathways, including base excision repair (BER), nucleotide excision repair (NER), homologous recombination (HR), and non-homologous end joining (NHEJ), that are activated during freeze–thaw stress to repair oxidative and strand break damage. Epigenetic regulators such as DNA methyltransferases (DNMTs), histone methyltransferases, and histone deacetylases (HDACs) dynamically remodel chromatin and modulate DDR signaling, facilitating efficient repair and transcriptional reprogramming during recovery. Comparative evidence from freeze-tolerant vertebrates, hibernating mammals, and polar fish underscores the conservation of these adaptive pathways across taxa. Integrating these insights provides a molecular network perspective (MNP) linking DDR and epigenetic mechanisms to environmental resilience, with potential applications in crop improvement and biotechnological adaptation strategies for extreme environments.

## 1. Introduction

Surviving in subzero temperatures requires organisms to overcome barriers linked to extreme physiological challenges, including oxidative stress, metabolic arrest, dehydration, and genomic instability [1]. Freeze-tolerant species ranging from amphibians and reptiles to insects and plants have evolved diverse adaptations that enable prolonged survival in cold environments [2,3,4]. These include the use of cryoprotectants, antioxidant defenses, metabolic rate depression, and tightly regulated systems for preserving genome integrity during freeze/thaw cycles [1,5,6]. Among the most critical threats to cellular survival during cold exposure is DNA damage, which arises from replication stress, reactive oxygen species (ROS), and mechanical disruption caused by ice crystal formation [7,8,9]. To maintain genome stability, freeze tolerant species activate DNA damage response (DDR) pathways in a uniquely timed manner suppressing repair during freezing to conserve energy and reactivating it upon thawing. This energy-efficient strategy ensures DNA repair is initiated only when conditions permit effective recovery [2,10].

Recent studies have shown an essential role for epigenetic regulation, particularly histone modifications, in controlling DDR in cold-stressed organisms. Histone marks such as H3K27me3 and H3K36me3 function as molecular switches, regulating the expression of repair genes and modifying chromatin accessibility [11,12,13]. In wood frogs and hibernating mammals (13 lined ground squirrel), changes in histone modifications that are believed to be associated with DDR activation, facilitating DNA repair during thawing [14,15,16]. These findings highlight a complex interplay between chromatin structure and genome maintenance under environmental stress [17,18]. Despite this progress, significant knowledge gaps remain in understanding how histone modifications and DDR are coordinated in cold-adapted species. Most existing studies focus on either cold-induced DNA damage or stress-related chromatin remodeling but rarely explore the integration of both processes. Questions remain regarding the timing, reversibility, and functional regulation of histone modifications across freezing and thawing phases and how these epigenetic marks regulate DDR pathways in vivo.

In this review, we first examine the physiological strategies that enable cold adaptation across taxa. We then explore the nature of cold-induced DNA damage, the molecular components of DDR, and the roles of histone modifications in regulating genome repair under cold stress. Finally, we discuss recent insights from molecular network analysis and identify future directions for research, including the need for integrative approaches to examine epigenetic control of DDR. We conclude by emphasizing critical gaps in the field. For instance, are new experimental tools needed to detect histone–DDR interactions in vivo under cold conditions? Can omics-based approaches capture the temporal and tissue-specific dynamics of these responses? Moving forward, the integration of molecular biology, chromatin profiling, and computational modeling will be essential to fully understand DDR regulation in cold-adapted species and to translate these findings into innovations in cryopreservation, climate resilience, and stress-adaptive biotechnology [3,4,19,20].

### 1.1. Cold-Adapted Species

Freeze tolerance is an extraordinary adaptation that enables certain vertebrates, such as amphibians and reptiles and even plants, to survive with up to 65% of their body water frozen for extended periods [2,19]. These organisms endure complete metabolic arrest but recover rapidly upon thawing. Their survival depends on two key strategies: cryoprotectant accumulation and controlled ice formation. Cryoprotectants, such as glucose and urea, mitigate intracellular dehydration and osmotic stress [6], while ice-binding proteins (IBPs) and aquaporins regulate water movement and prevent lethal ice crystal formation [1,5]. In addition to cryoprotectants, metabolic rate depression, antioxidant defence systems and anti-apoptotic response are other mechanisms that these organisms use to survive extreme cold conditions [1,2]. Furthermore, recent studies have also begun to uncover the epigenetic regulation of gene expression as a crucial component of cold survival. Histone modifications, including both repressive and activating marks, have been observed in freeze-tolerant species like the wood frog and are thought to control the transcription of genes involved in stress responses, metabolism, and DNA repair [5,13,16,21]. Table 1 summarizes key freeze-tolerant organisms and the known histone modifications and stress responses associated with their cold adaptation strategies. These examples illustrate the diversity of species studied and highlight the emerging relevance of epigenetic and genomic maintenance in surviving extreme cold. However, beyond the immediate mechanical challenges of freezing, these organisms must also maintain genomic integrity in the face of extreme environmental stressors, particularly oxidative stress and DNA damage caused by freeze–thaw cycles.

### 1.2. DNA Damage in Cold Stress

Organisms exposed to extreme cold environments face significant physiological and biochemical challenges [2], with one of the most critical being DNA damage. Cold/freezing stress, particularly in species that experience the freeze/thaw cycles, leads to the accumulation of genotoxic stressors, including oxidative stress, mechanical damage from ice crystal formation, and replication-associated stress [7,8,33,34]. If left unrepaired, these DNA lesions can compromise genomic integrity, affecting cellular survival and adaptation [35]. Cold-adapted species have evolved highly efficient DDR pathways to counteract these effects, ensuring that DNA repair mechanisms are tightly coordinated with stress adaptation strategies [8].

Cold-induced DNA damage arises from multiple mechanisms. One of the most immediate threats is mechanical damage caused by the formation of ice crystals within cells and tissues [2]. As water freezes, it expands, leading to physical stress that disrupts cellular structure, including chromatin structure and nuclear membranes [9]. This stress can result in double-strand breaks (DSBs), chromatin fragmentation, and telomere instability, increasing the risk of genomic rearrangements. In the wood frog, a freeze-tolerant species controlled ice formation occurs in extracellular spaces, minimizing intracellular damage [7,8]. However, some degree of DNA damage is inevitable and requires subsequent repair. Another significant contributor to DNA damage during cold stress is oxidative stress, which occurs due to the overproduction of reactive oxygen species (ROS) [2,8]. During freezing, metabolic rates decline, leading to hypoxic conditions within cells. Upon thawing, reoxygenation triggers a surge in mitochondrial activity, resulting in increased ROS production [8]. These ROS interact with DNA, causing oxidative base modifications such as 8-oxo-7,8-dihydroguanine (8-oxoG) [36], single-strand breaks (SSBs), and lipid peroxidation byproducts that form mutagenic DNA adducts [36,37]. If not repaired efficiently, oxidative DNA damage can lead to permanent mutations and genomic instability [38]. Species like the Arctic ground squirrel, which undergo extended periods of hibernation and metabolic suppression [39,40], rely on enhanced antioxidant defenses and DNA repair pathways to counteract this oxidative stress upon arousal from torpor [39]. In addition to mechanical and oxidative damage, replication stress poses a challenge for cells that attempt to divide under cold conditions. Across several species, low temperatures stall or arrest cell cycle progression, preventing replication-associated DNA damage [40,41,42,43]. However, for actively dividing cells, cold stress can stall replication forks, leading to collapse of the replication fork, incomplete DNA synthesis, and accumulation of DSBs [43]. Cold-induced replication errors increase the risk of polymerase slippage and insertion/deletion mutations [44]. Certain plants like *Arabidopsis thaliana*, cold stress is known to control the expression of DNA replication and repair genes, ensuring that stalled replication forks are stabilized and repaired to prevent genomic instability [45,46].

The extent of DNA damage in cold-adapted species is influenced by their freeze-tolerance strategies. Some organisms, such as freeze-tolerant amphibians and insects, allow ice formation in extracellular and extra-organ spaces while protecting intracellular components using cryoprotectants like trehalose and glucose, which help stabilize cellular structures and reduce dehydration-associated DNA damage [30,31,32,47,48]. Other species, particularly freeze-avoidant organisms like certain fish and overwintering insects, rely on antifreeze proteins (AFPs) and supercooling strategies to prevent ice formation altogether, thereby minimizing direct mechanical damage to DNA [10].

One of the key differences between cold-adapted species and non-adapted species is the timing of DNA repair activation. Many freeze-tolerant species exhibit a delayed DNA repair response, where repair pathways remain largely inactive during freezing but become highly active upon thawing [10,30]. This strategy ensures that ATP and repair factors are not expended under energy-limited conditions but are instead deployed efficiently during the post-thaw recovery phase [1,2]. For example, in the model freeze-tolerant organism, the wood frog, DDR activation is marked by the phosphorylation of histone H2AX (γH2AX) upon thawing, indicating the recruitment of repair complexes to sites of DNA damage [8]. This delayed yet coordinated response highlights the complex relationship between DDR and cold adaptation.

Cold-induced DNA damage has broader implications for genomic stability and evolutionary adaptation. The persistence of DNA lesions in extreme environments may drive the selection of enhanced DDR efficiency and epigenetic regulatory mechanisms that contribute to long-term genomic stability. In particular, histone modifications such as H3K3me3 and H3K27me3 play a crucial role in controlling the expression of DDR genes and maintaining chromatin integrity under cold stress [3,12]. These modifications not only regulate repair processes but also serve as an epigenetic memory of cold exposure, enabling organisms to mount a more rapid and efficient response to subsequent freeze–thaw cycles.

While subzero temperatures and stress impose significant challenges to DNA integrity, primarily through mechanical disruption, oxidative damage, and replication stress, cold-adapted species have evolved specialized mechanisms to lessen these effects, utilizing DDR pathways, antioxidant defenses, and epigenetic regulation to ensure genomic stability.

## 2. DNA Damage Response

DNA is continuously subjected to various types of damage, such as mutations, oxidative stress, replication errors, and environmental insults, including temperature changes, radiation and chemicals [49,50]. If left unrepaired, this damage can lead to mutations, chromosomal instability, and complex diseases like cancer and neurological diseases [50]. The DDR is a fundamental cellular mechanism that detects, signals, and repairs DNA damage to maintain the integrity of the genome [51]. The DDR is a highly coordinated network of pathways designed to protect cells from such detrimental effects, ensuring proper cell division, functioning, and overall organism health [38,52].

DNA damage can be broadly classified into two categories based on the nature of the lesion: base damage and structural damage [53]. Base damage involves the modification of individual DNA bases, which can result from chemical reactions or oxidative stress. This includes oxidative lesions, deamination (e.g., cytosine to uracil), alkylation, and pyrimidine dimers (from UV radiation) [54,55]. For example, oxidative stress can convert guanine to 8-oxo-7,8-dihydroguanine, which mis-pairs with adenine during replication [36,55]. Structural damage includes more severe forms of DNA damage, such as single-strand breaks (SSBs) and double-strand breaks (DSBs) [56]. Single-strand breaks are less severe than double-strand breaks but can still cause problems if left unrepaired. Double-strand breaks, where both strands of the DNA helix are severed, are the most dangerous form of DNA damage, as they can lead to chromosomal fragmentation, translocations, and loss of genetic information [57]. Additionally, chemical agents like those used in chemotherapy can cause DNA crosslinking, preventing replication and transcription. Replication stress, often caused by stalled replication forks, can also result in DSBs [38].

The DDR consists of three major stages: damage detection, signal transduction, and repair, which together ensure that DNA damage is either repaired accurately or that cells undergo apoptosis if the damage is irreparable. The first step, damage detection, is facilitated by several key sensor proteins. The MRN complex (Mre11-Rad50-Nbs1) is involved in detecting DSBs and forms the basis for the early response, processing DSBs and initiating repair mechanisms such as homologous recombination (HR) [58]. ATM (Ataxia Telangiectasia Mutated) is activated by the MRN complex in response to DSBs and plays a central role in signaling pathways that lead to cell cycle arrest and DNA repair [59,60]. ATR (ATM and Rad3-related) responds to single-strand DNA or replication stress and is activated by the presence of single-strand regions of DNA, such as those formed at stalled replication forks [61]. DNA-PKcs, part of the non-homologous end joining (NHEJ) repair pathway, is responsible for detecting this DSBs [62]. Recent studies in freeze-tolerant vertebrates and hibernating mammals have begun to reveal the activation of specific DNA repair pathways during post-freeze recovery. In *R. sylvatica*, markers of base excision repair (BER), such as upregulation of *OGG1* and *APE1*, have been detected following thawing, indicating targeted repair of oxidative lesions generated during reoxygenation [8]. Similarly, hibernating Arctic ground squirrels display the induction of homologous recombination (HR) proteins including RAD51 and BRCA1, suggesting a conserved role of HR in repairing double-strand breaks post-hibernation [40].

After DNA damage is detected, it must be transmitted to the cellular machinery to coordinate the repair response. This is achieved through the activation of signaling proteins, including kinases like ATM and ATR, which initiate the DDR cascade by phosphorylating downstream targets, including histones, repair proteins, and checkpoint proteins. One notable modification is the phosphorylation of histone H2AX at serine 139, forming γH2AX, which marks the site of DNA damage and attracts repair factors [63,64]. Additionally, checkpoint kinases like Chk1 and Chk2 are activated by ATM and ATR to halt the cell cycle, ensuring that the cell does not proceed to mitosis until the damage is repaired. These checkpoint proteins phosphorylate cell cycle regulators, such as cyclin-dependent kinases, to prevent the cell from entering mitosis with unresolved DNA damage [38]. The repair stage involves fixing the DNA lesions detected in the first step. Depending on the type of damage, the cell utilizes different repair pathways. Base excision repair (BER) repairs small, non-helix-distorting base modifications caused by oxidative stress and chemical damage by excising the damaged base and replacing it with the correct one [65]. Nucleotide excision repair (NER) is used to repair bulky, helix-distorting lesions such as pyrimidine dimers induced by UV radiation, where a segment of DNA containing the damage is excised and replaced with the correct sequence [66]. Mismatch repair (MMR) corrects base mispairing and small insertion/deletion loops that occur during DNA replication by recognizing the damaged strand and excising the incorrect nucleotide [67]. Homologous recombination (HR) uses a homologous DNA sequence as a template to repair DSBs [68]. Non-homologous end joining (NHEJ) is an error-prone repair pathway that directly ligates the ends of broken DNA, typically used for DSBs in non-dividing cells when a homologous template is not available [67].

DDR is closely tied to the regulation of the cell cycle. Cell cycle checkpoints are activated to allow the cell to suspend its cycle and repair the damage before continuing [69]. The main checkpoints involved in DDR include the G1/S checkpoint, which ensures that DNA is intact before the S phase begins, preventing the replication of damaged DNA. The S-phase checkpoint monitors the integrity of replication forks and, if replication stress occurs, stalls DNA replication to allow time for repair. Finally, the G2/M checkpoint prevents cells from entering mitosis with unresolved DNA damage. If the damage is not repaired, the cell is prevented from dividing.

Because DDR is highly regulated and coordinated, several factors work together to maintain genome stability. These factors include sensor proteins, signal transducers, effectors, and regulatory networks. In addition, recent studies have shown that epigenetic modifications play a role in regulating DDR [18,70,71]. These epigenetic modifications include histone modifications, DNA methylation, and chromatin remodeling and they all play critical roles in how DDR is coordinated and how cells respond to DNA damage.

### 2.1. Histone Modifications

Histone modifications play a pivotal role in the adaptation of cold-adapted species by regulating chromatin structure, controlling gene expression, and facilitating DDR pathways to counteract freezing-induced stress. These modifications, which include acetylation, methylation, phosphorylation, and ubiquitination, serve as molecular switches that modify transcriptional and repair programs, ensuring cellular resilience and genomic stability in extreme environments [17,70,72]. Histone acetylation, a well-studied modification typically leads to chromatin relaxation [73]. This relaxed chromatin structure enhances the accessibility of DNA repair proteins, facilitating the repair of damage by making the underlying DNA more accessible to the repair machinery [17]. Acetylation of histone residues such as H3K9ac, H3K27ac, and H4K16ac has been specifically shown to promote DNA repair [74]. These acetylated histone marks are often associated with active transcription and DNA repair sites, supporting the recruitment of repair factors to areas of damage [74,75]. The acetylation process removes positive charges on the histones, resulting in a more open chromatin configuration that allows for the efficient recognition and repair of damaged DNA [75]. For instance, In *Arabidopsis thaliana* and some cold resistant plants, acetylation at H3K9ac and H4K16ac, has been shown to promote the transcription of cold-responsive genes [4,76]. This chromatin relaxation facilitates the rapid activation of stress–response pathways, allowing plants to adjust their metabolism and repair cold-induced damage efficiently. Additionally, in the wood frog brain and kidney, reversible histone acetylation was shown to be essential in regulating gene expression and facilitating DNA repair during the freezing and thawing cycles, enabling their survival under extreme cold stress [11,22]. This is concurrent with findings in hibernating mammals like the 13 lined ground squirrel [16].

In contrast to acetylation, histone methylation plays a more complex role in the regulation of DNA repair and chromatin structure. The methylation of H3K9me3 is particularly significant, as it is linked to gene silencing and heterochromatin formation. H3K9me3, an essential marker of heterochromatin [77], is involved in maintaining the structural integrity of regions that are transcriptionally inactive, such as centromeres and telomeres, which are critical for preventing genomic instability [77]. The formation of heterochromatin through methylation processes can play a protective role by suppressing repetitive DNA sequences that are prone to DNA damage or recombination [78,79]. This silencing mechanism is essential for maintaining genomic stability, as it minimizes the risk of damage accumulation in repetitive regions, which are often hotspots for genomic instability and mutations. For example, *Rana sylvatica*, histone methylation plays a dual role in gene regulation. The presence of H3K27me3, a well-known transcriptionally repressive mark, helps suppress non-essential genes during freezing to conserve energy, while H3K36me3 is upregulated post-thaw to activate DNA repair genes, ensuring rapid genome restoration [11,13,15]. Similarly, in hibernating mammals such as the Arctic ground squirrel and the 13 lined ground squirrel, the persistence of histone methylation marks like H3K36me3 at DDR loci ensures efficient genome repair following repeated freeze–thaw cycles, reducing long-term genomic instability [16,40]. This epigenetic memory enhances survival fitness in species that experience seasonal freezing, providing a selective advantage in harsh environments. Histone methylation not only regulate gene expression in response to cold stress but also contribute to an “epigenetic memory” of past exposures, allowing organisms to mount a faster and more efficient response to subsequent cold events. As seen in *Arabidopsis thaliana*, H3K27me3 persists at cold-stress genes even after temperatures return to normal, priming these genes for quicker reactivation upon future exposure to freezing conditions [3,12].

Phosphorylation of histones, particularly histone H2AX at serine 139 (forming γH2AX), is a hallmark modification in the DDR, playing a key role in the repair of DNA double-strand breaks (DSBs), one of the most lethal forms of DNA damage [80]. γH2AX rapidly accumulates at the sites of DSBs, marking these regions as sites of active repair [80,81]. This modification serves as a recruitment platform for various repair proteins, including the MRN complex (Mre11-Rad50-Nbs1), 53BP1, and others involved in both homologous recombination and non-homologous end joining repair pathways. The formation of γH2AX is one of the earliest detectable events in the DDR and facilitates the assembly of repair complexes that are essential for maintaining genome integrity after severe DNA damage [81]. DSB repair has been observed in freeze-tolerant amphibians and hibernating mammals, where its accumulation post-thaw correlates with the recruitment of repair proteins such as ATM, 53BP1, and BRCA1 [8]. While not much studies have been done on phosphorylation, it is believed that rapid phosphorylation response is essential for preserving genome integrity following cold-induced DNA damage.

Knowledge of histone modification-mediated DDR pathways in cold-tolerant species may contribute to the development of more effective cryopreservation techniques, improving the storage and viability of biological materials such as human tissues, cells, and organs.

### 2.2. Molecular Network Analysis

Molecular network analysis is a powerful approach that integrates high-throughput genomic, transcriptomic, and proteomic data to reveal the interactions between genes, proteins, and cellular pathways, particularly in the DDR and histone modifications in freeze tolerant organisms. Although cold-adapted species have evolved numerous biochemical defenses, the regulation of DDR by histone modifications remains poorly mapped at the systems level. High-throughput technologies such as RNA-Seq, ChIP-Seq, and proteomics can be applied to identify histone-modifying enzymes (e.g., HATs, HDACs, HMTs, HDMs) involved in DDR gene regulation, co-expression networks linking cold-responsive transcription factors to chromatin remodelers, temporal patterns of histone mark dynamics across freeze–thaw cycles, DDR protein–protein interaction networks in tissues experiencing cold-induced genotoxic stress.

In plants like *Arabidopsis thaliana*, modifications on H3K4me3 and H3K27me3 control the DNA repair pathways to maintain genomic stability during freezing conditions [3,12]. These marks are associated with chromatin “memory”, allowing plants to respond more quickly to repeated cold exposure [82]. ChIP-Seq analyses have confirmed that cold stress alters the genome-wide distribution of these marks, particularly at loci involved in DNA repair, oxidative stress response, and chromatin remodeling [3]. Similarly, metabolomics and transcriptomics analysis on rice has shown that environmental stress caused by low temperatures led to the expression of *OsHPL1*, a CYP74 family member, which caused the accumulation of 12-oxo-phytodienoic acid and jasmonates [83]. Furthermore, another comprehensive omics analysis on *Dendrobium officinale* revealed that the ubiquitination proteins PKU64802, XP_020672210, and PKU75555 were found to regulate splicing factors, which showed increased abundance under cold stress conditions [19]. These findings demonstrate that cold stress affects not just DNA and histones, but entire signaling cascades providing multiple regulatory entry points for DDR control.

While there are not many studies to show the molecular network analysis in freeze-tolerant animals, we believe that the integration of molecular network analysis in studying cold adaptation will provide more understandings into how these species survive extreme environmental stress and offers potential applications in biotechnology, including advancing cryopreservation techniques in humans. Future work could benefit from combining network modeling tools like the WGCNA, Cytoscape, STRING with experimental approaches such as ATAC-Seq for assessing chromatin accessibility and CUT&RUN used to profile histone marks with low input [84,85,86,87,88]. Such integration would enable precise mapping of how chromatin state influences DDR, and how epigenetic responses are temporally controlled under cold stress.

## 3. Conclusions

Cold-adapted species have evolved an extraordinary set of molecular tools to survive extreme environmental conditions that would otherwise compromise genomic stability. At the heart of these adaptations is a highly regulated DDR, which functions in a temporally coordinated manner delayed during freezing to conserve energy and activated upon thawing to repair accumulated lesions. This precise regulation ensures the maintenance of genome integrity in the face of oxidative damage, replication stress, and chromatin disruption caused by freezing and reoxygenation. Central to this regulation is the role of histone modifications, which act as epigenetic switches controlling chromatin structure, gene expression, and the recruitment of DNA repair machinery. Acetylation promotes chromatin accessibility and repair enzyme recruitment, while methylation dynamically represses or activates specific gene sets depending on the organism’s metabolic state. The interplay between these modifications creates a flexible yet stable system that allows freeze-tolerant species to silence and resume key biological and metabolic processes as needed. Furthermore, the persistence of certain histone marks following cold exposure suggests the existence of an epigenetic memory, enabling a more rapid and efficient response to recurring stress. Despite significant advances, much remains to be understood about how DDR and histone modifications interact in cold-adapted systems. To address these research gaps, the field must embrace multi-omics approaches and molecular network analysis. By integrating transcriptomics, epigenomics, proteomics, and metabolomics, researchers can begin to map the full architecture of cold stress responses. Such work would benefit from advanced tools like ChIP-Seq, CUT&RUN, ATAC-Seq, and computational modeling, which can reveal dynamic, tissue-specific interactions between chromatin states and DDR components. From a translational perspective, insights from cold-adapted species hold significant potential. Better understanding of histone-mediated DDR could inform cryopreservation technologies, improve tissue and organ storage, and guide the development of stress-resilient crops. These findings may also have biomedical applications, such as in hypothermia therapy, radiation protection, or enhancing genome stability in stem cells. Finally, cold-adapted species provide a natural model system for studying the intersection of environmental stress, chromatin biology, and DNA repair. Unraveling these connections will not only deepen our understanding of evolutionary resilience but also provide novel strategies for enhancing biological preservation, agriculture, and human health in the face of environmental and clinical stressors.

## Figures and Tables

**Table 1:** Freeze-Tolerant Organisms and Their Epigenetic and DNA Damage Response Mechanisms.

Organism	Studied Mechanisms	References
Wood frog (*Rana sylvatica*)	Histone methylation (H3K27me3, H3K36me3), histone acetylation, antioxidant response, DNA repair post-thaw	Bloskie et al., 2024 [11]; Lung & Storey, 2022 [8]; Taiwo et al., 2024 [15]; Taiwo et al., 2025 [22]
Arctic ground squirrel (*Urocitellus parryii*)	Antioxidant defenses, lack of cellular stress post-hibernation	[23,24,25]
13-lined ground squirrel *(Ictidomys tridecemlineatus*)	Reversible histone acetylation and methylation during torpor	[16]
Boreal chorus frog (*Pseudacris maculata*)	Accumulation of cryoprotectants (glucose, urea), freeze tolerance mechanisms	[26]
Painted turtle (*Chrysemys picta*)	Anoxia tolerance, metabolic depression, DNA repair mechanisms	[1,27]
Antarctic Icefish (*Chionodraco hamatus*)	Histone modifications under cold stress	[28,29]
Overwintering insects (e.g., *Eurosta solidaginis*)	AFP production, cryoprotectants (glycerol, trehalose), metabolic reprogramming	[30,31]
Eco-dormant birch buds (*Betula* spp.)	Structural freeze resistance, vitrification, antioxidant systems	[32]

## Data Availability

No new data were created or analyzed in this study. Data sharing is not applicable to this article.
